# Hantavirus Pulmonary Syndrome in Santa Cruz, Bolivia: Outbreak Investigation and Antibody Prevalence Study

**DOI:** 10.1371/journal.pntd.0001840

**Published:** 2012-10-18

**Authors:** Joel M. Montgomery, Patrick J. Blair, Darin S. Carroll, James N. Mills, Alberto Gianella, Naomi Iihoshi, Ana M. Briggiler, Vidal Felices, Milagros Salazar, James G. Olson, Raisa A. Glabman, Daniel G. Bausch

**Affiliations:** 1 Special Pathogens Branch, Centers for Disease Control and Prevention, Atlanta, Georgia, United States of America; 2 U.S. Naval Medical Research Unit 6, Lima, Peru; 3 Bolivian National Center for Tropical Diseases, Santa Cruz, Bolivia; 4 Argentine National Institute for Viral Diseases “Dr. Julio I. Maiztegui”, Pergamino, Argentina; 5 Department of Tropical Medicine, Tulane School of Public Health and Tropical Medicine, New Orleans, Louisiana, United States of America; University of Texas Medical Branch, United States of America

## Abstract

We report the results of an investigation of a small outbreak of hantavirus pulmonary syndrome in 2002 in the Department of Santa Cruz, Bolivia, where the disease had not previously been reported. Two cases were initially reported. The first case was a physician infected with Laguna Negra virus during a weekend visit to his ranch. Four other persons living on the ranch were IgM antibody-positive, two of whom were symptomatic for mild hantavirus pulmonary syndrome. The second case was a migrant sugarcane worker. Although no sample remained to determine the specific infecting hantavirus, a virus 90% homologous with Río Mamoré virus was previously found in small-eared pygmy rice rats (*Oligoryzomys microtis*) trapped in the area. An antibody prevalence study conducted in the region as part of the outbreak investigation showed 45 (9.1%) of 494 persons to be IgG positive, illustrating that hantavirus infection is common in Santa Cruz Department. Precipitation in the months preceding the outbreak was particularly heavy in comparison to other years, suggesting a possible climatic or ecological influence on rodent populations and risk of hantavirus transmission to humans. Hantavirus infection appears to be common in the Santa Cruz Department, but more comprehensive surveillance and field studies are needed to fully understand the epidemiology and risk to humans.

## Author Summary

Hantaviruses can evoke a severe, acute disease in humans known as hantavirus pulmonary syndrome with case fatalities up to 70%. Pathogenic hantaviruses are carried by rodents, with each virus species usually carried by a specific species of rodent. Hantavirus-host reservoir pairs continue to be discovered and details of the epidemiology and risk of hantaviruses to humans continue to emerge. We report the results of an investigation of a small outbreak of hantavirus pulmonary syndrome in 2002 in the Department of Santa Cruz, Bolivia, where the disease had not previously been reported. Two cases were initially noted, with four additional persons shown to be recently infected with hantaviruses through thorough field investigation and antibody evidence. An antibody prevalence study conducted as part of the outbreak investigation showed over 9% of the population studied to have previous exposure to hantaviruses. Precipitation in the months preceding the outbreak was particularly heavy in comparison to other years, suggesting a possible climatic influence on rodent populations and risk of hantavirus transmission to humans. Hantavirus infection appears to be common in the Santa Cruz Department, but more comprehensive surveillance and field studies are needed to fully understand the epidemiology and risk to humans.

## Introduction

Hantaviruses (family *Bunyaviridae*, genus Hantavirus) are enveloped, tripartite, single-stranded, negative-sense RNA viruses. On the American continents, these viruses can evoke a severe, acute disease in humans known as hantavirus pulmonary syndrome (HPS) [1]. Hantavirus pulmonary syndrome is characterized by fever, headache, myalgia, and, in severe cases, rapid cardiopulmonary dysfunction, with case fatalities up to 70% depending on the particular virus. Hantaviruses are maintained in rodents and insectivores, usually with a tight pairing between the specific virus and host species. All hantaviruses known to cause human disease are associated with rodent hosts. Since the first recognized case of HPS in 1993, an estimated 200 cases per year associated with more than 25 different hantaviruses have been recognized in the Americas, the majority in South America [1]. Sin Nombre virus in North America and Andes, Araraquara, and Laguna Negra (LANV) viruses in South America are among the most frequent etiologic agents. Antibody-prevalence studies in some area of South America suggest hantavirus exposure in up to 40% of the population [2]. Furthermore, hantavirus-host reservoir pairs continue to be discovered and details of the epidemiology and risk of hantaviruses to humans continue to emerge.

**Figure 1:**
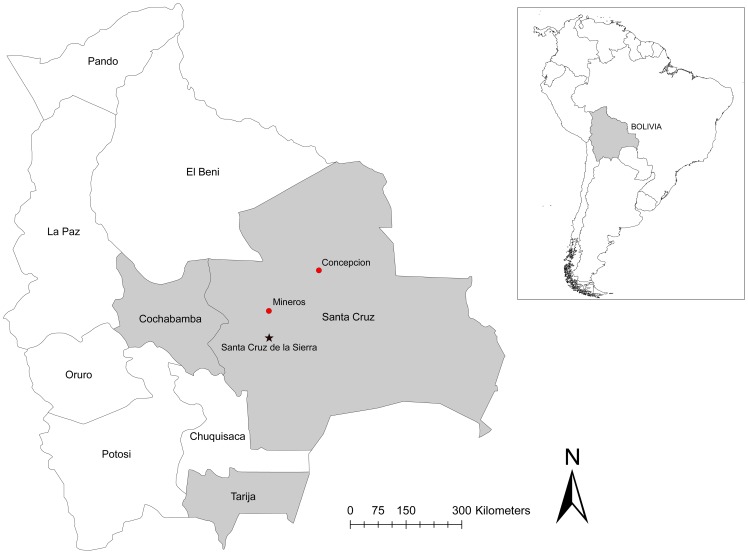
Locations of reported cases of hantavirus pulmonary syndrome and field studies associated with the 2002 outbreak in Bolivia. The two index cases reported here were from Mineros and Concepción. The capital city of Santa Cruz is indicated by a star. Laboratory-confirmed hantavirus infection in humans and rodents has been reported from Santa Cruz, Tarija, and Cochabamba Departments, shown in gray.

Between May and June, 2002, the Bolivian National Center for Tropical Diseases (CENETROP) reported HPS in two residents of geographically disparate areas of the Department of Santa Cruz, Bolivia (Figure 1). Because HPS had not been previously recognized in Santa Cruz, a multinational effort was undertaken in August 2002 to 1) assess the circumstances surrounding these cases, 2) clarify the public health risk posed by hantaviruses in the region, and 3) characterize the virus-reservoir pairing(s). Results of the investigation of the rodents implicated in the outbreak have been previously reported [3]. Here we report the results on the human cases and ancillary epidemiological studies conducted as part of the outbreak investigation.

## Materials and Methods

### Ethics statement

The activities in which the human samples were taken were approved by the U.S. Naval Medical Research Center in compliance with all applicable Federal regulations governing the protection of human subjects. All subjects provided informed oral consent. Oral rather than written consent was chosen because the literacy level of the population was estimated to be low (less than 30%). Oral consent was documented by two witnesses, one from the study team and one from family members or friends present at the time of the interview and blood draw. Parents or guardians provided consent on behalf of all minors, with the assent of the minor. The consent process and all other activities reported in this manuscript were approved by CENETROP; the U.S. Centers for Disease Control and Prevention, Atlanta, USA; U.S. Naval Medical Research Unit 6, Lima, Peru; and Argentine National Institute for Viral Diseases “Dr. Julio I. Maiztegui”, Pergamino, Argentina as components of the emergency response to a hantavirus outbreak occurring in the Department of Santa Cruz, Bolivia, in 2002. Samples tested from already-existing collections at CENETROP were numerically coded, with the identity of the person available only to clinicians interacting with the patient.

### Case reports

#### Case 1

On May 3, 2002, an otolaryngologist working at a hospital in the city of Santa Cruz fell sick with fever, myalgia, and headache followed a few days later by dry cough. His symptoms worsened and he was hospitalized on May 10, 2002, where a chest X-ray showed bilateral noncardiogenic pulmonary edema. His condition gradually improved with supportive therapy over the following weeks and he was discharged in good condition on May 28, 2002.

This confirmed case of HPS in a healthcare worker initially raised suspicion of nosocomial transmission, especially in light of reports of person-to-person transmission of hantaviruses in South America [4]. However, the physician denied any exposure in his clinical practice to persons with febrile or respiratory diseases. Further investigation revealed that he owned a cattle ranch near Concepción, approximately 200 Km northeast of the city of Santa Cruz, where he and his wife would often spend weekends (Figure 1). The area, once densely forested, is now a mix of forest and cleared land for cattle pasture and human dwellings, which are primarily rustic adobe houses with thatched roofs. There are a few such houses on the physician's ranch, one of which he and his wife occupied during their visits. Rodents were frequently noted in and around the premises.

The physician's last reported visit to the ranch was March 29–31, 2002, 33–36 days prior to onset of his illness. On two occasions, including during this visit, while in bed at night he heard animals (presumably rodents, although he could not see the animals in the darkness) running on the rafters above and felt drops of (presumably) urine falling onto his face. The physician also reported that one of his farm-hands had a similar disease to his about 2 months prior, although milder and not requiring hospitalization.

#### Case 2

Few details were available regarding the second case who was a sugarcane worker living in the make-shift tent villages on the periphery of the sugarcane fields near the town of Mineros, approximately 100 Km north of the city of Santa Cruz (Figure 1). He fell sick with a febrile illness on June 30, 2002. On July 7, 2002, he sought medical care at a local clinic but, due to his severe condition, was immediately transferred to a hospital in Santa Cruz, where he died 5 hours after admission. Like many of the sugarcane workers in the area, the patient was a migrant, having come to Mineros with his wife and child from the Department of Cochabamba. His family left the area after he died and was not available for interview or testing. Although we were unable to determine the exact dates in which he worked in the Mineros sugarcane fields, the crew to which he belonged had been working there for some months, suggesting that he was infected there.

There was no contact or apparent link between the cases in Concepción and Mineros. Serum samples from both patients were sent to CENETROP where they were found to be IgM antibody-positive by enzyme-linked immunosorbent assay (ELISA) using a LANV antigen, indicating acute infection with a hantavirus [5]. Subsequent RT-PCR amplification and sequencing of a 434 nucleotide segment of the nucleocapsid coding region of the S segment showed the virus infecting the physician to be 88% homologous with LANV [6]. No sample remained from the sugarcane worker for PCR or sequencing. CENETROP received unconfirmed reports of at least two additional suspected cases of HPS-like illness in the area but, again, no samples were available for testing.

### Antibody-prevalence studies

Cross-sectional antibody-prevalence studies, approved by the Bolivian Ministry of Health, were conducted in Mineros and Concepción to elucidate the transmission dynamics in this outbreak as well as the overall risk of hantavirus infection in the region. After obtaining informed consent, 5 ml of blood were collected from a convenience sample of persons aged ≥5 years. In addition, a one-page questionnaire addressing potential risk factors for hantavirus infection and types of exposure was administered in Spanish to each study participant.

In Mineros, three communities (Dinamarca, La Patria, and Oriental) were selected based on the hypothesis that exposure to rodents and hantaviruses would be higher in communities actively involved in the sugarcane harvest. Dinamarca consisted of approximately 50 dwellings (4–5 persons/house) constructed of plastic tarps on the periphery of the sugarcane fields. This community migrates frequently to follow the sugarcane harvest. The male inhabitants work in the fields while women and children typically attend to domestic chores. La Patria is a permanent town of approximately 60–80 inhabitants, mostly subsistence farmers of rice and vegetables, on the outskirts of the sugarcane fields. Oriental is an urban neighborhood on the edge of Mineros, outside of the sugarcane growing area, whose inhabitants work in diverse occupations in and around Mineros, with only a few persons working in the sugarcane fields. In Concepción, blood was collected from persons living on the physician's ranch and in surrounding communities.

Testing was performed at CENETROP with assistance from U.S. Naval Medical Research Unit 6 from Lima, Peru, following standard operating procedures supplied by the Centers for Disease Control and Prevention. All samples were tested by ELISA for IgG antibody to hantavirus using a previously described methodology [5]. Because there were unconfirmed reports of recent cases of HPS in Concepción, samples collected from this area were also tested for IgM antibody. The antigens used for the IgG and IgM assays were from Sin Nombre virus and LANV, respectively, which have been shown to be broadly cross-reactive with New World hantaviruses. The starting dilution was 1∶100 with subsequent 4-fold dilutions. Conservative cut-offs for a positive result were used—optical densities of >3.0 for the IgG assay and >2.0 for IgM.

## Results

**Figure 2:**
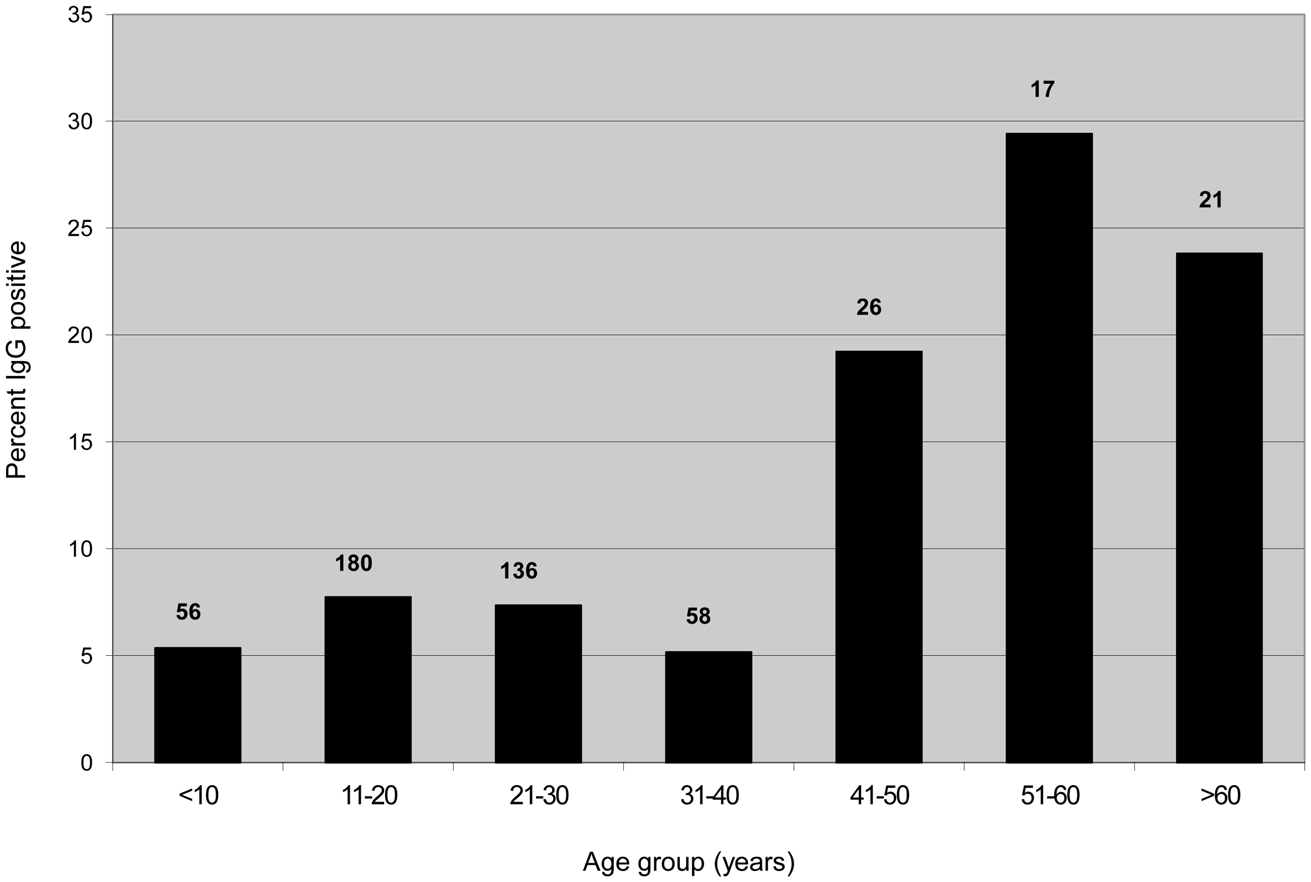
Hantavirus IgG antibody prevalence by age group in Mineros and Concepcion, Bolivia, 2002. Numbers on top of the bars indicate the total number of persons tested in that age group.

A total of 494 persons were enrolled in the serosurvey, 415 from Mineros and 79 from Concepcion (Table 1). The mean age was 21 years (range 5–81) and 62% were male. The overall IgG antibody prevalence in all regions was 9.1% and did not differ significantly between any of the towns or communities studied. IgG-positive cases were noted in 35 households, with seven households having two cases (five in Dinamarca and one each in La Patria and Oriental) and one household in Concepcion having four cases. There were no significant differences in the frequency of IgG antibody in persons with respect to sex, occupation, house construction materials, hunting or fishing, home department in Bolivia, time spent living or working in Santa Cruz Department, rural or urban house location, or noting the presence of rodents at home or at work. Antibody-positive persons were significantly older than antibody negative ones (33 versus 24 years, respectively, p = .006). The IgG antibody prevalence stratified by age group is shown in Figure 2. Although not statistically significant, the highest IgG prevalence was noted in persons with professions that would likely put them at increased risk of exposure to rural rodents, such as farmers (15.2%) and sugarcane workers (9.4%), as opposed to housewives (7.2%) and students/children (7.1%).

**Table 1:** Hantavirus antibody prevalence by ELISA in Mineros and Concepción, Bolivia, 2002.

Location	Number tested	Number IgG positive (%)^1^	Number IgM positive (%)^2^
**Mineros**			
Dinamarca	213	19 (8.9)	nd
La Patria	67	5 (7.5)	nd
Oriental	135	13 (9.6)	nd
**All Mineros**	(415)	37 (8.9)	nd
**Concepción**	79	8 (10.1)	4 (5.1)
**All Sites**	494	45 (9.1)	4 (0.8)

1 Mean optical densities on ELISA for the positive and negative samples were 0.943 (range 0.301–1.727) and 0.086 (range 0.000–0.298), respectively, at titers of 1∶100.

2 Mean optical densities on ELISA for the positive and negative samples were 0.431 (range 0.237–0.590) and 0.037 (range 0.000–0.142), respectively, at titers of 1∶400.

Abbreviations: ELISA-enzyme-linked immunosorbent assay; nd-not done.

The study population reported living in or coming from eight of Bolivia's nine departments, as well as from out of the country. However, IgG antibody-positive persons were found only from Beni (5/22, 22.7%), Cochabamba (3/15, 20.0%), Chuquisaca (14/134, 10.4%), Santa Cruz (20/245, 8.2%), and Potosí (3/57, 5.3%). No positive persons were noted from La Paz, Oruro, or Tarija, but there were only 16 people collectively from these departments, as well as six antibody-negative persons from outside the country. Interestingly, 29 (64%) of the 45 IgG antibody-positive persons had lived at their present residence for 1 year or less, suggesting that many hantavirus exposures may have occurred elsewhere, although the previous residence of this highly mobile population may often have been in the same region.

Four (5.1%) persons in Concepcion were IgM antibody-positive, all of whom lived and worked on the physician's ranch for a year or more. Three of the four cases, a ranch hand and his wife and 9-year-old daughter, clustered in a single household. These three persons, as well as a fourth member of the household, were also IgG-positive. The fourth IgM-positive person was a farmer. All IgM-positive persons were asymptomatic at the time of testing, but two reported febrile diseases accompanied by headache and myalgia in May and June, 2002, for which they did not seek medical care.

## Discussion

The cases described here are the first reported laboratory-confirmed cases of HPS in the Department of Santa Cruz, Bolivia. The sequence of the LANV from the physician was 99% identical to viruses obtained from a large vesper mouse (*Calomys callosus*) caught on his ranch, clearly marking this area as endemic for LANV [3]. This is further evidenced by the finding of other IgM-positive persons on the ranch, some of whom recently had syndromes consistent with mild HPS. Although the small vesper mouse (*Calomys laucha*) is considered the primary reservoir of LANV, the virus has been found in large vesper mice in Argentina, suggesting that LANV is also adapted to this rodent species [7]. Laguna Negra virus was also isolated from a Chilean traveler who developed HPS after traveling throughout Bolivia, including Santa Cruz Department [8]. The precise location of his infection is unknown. Hantavirus pulmonary syndrome due to LANV or LANV-like viruses has also been confirmed in Paraguay [6], Brazil [9], and Argentina [10].

Unfortunately, no sample was available to identify the specific virus that infected the sugarcane worker. However, a virus with 90% nucleotide homology with the hantavirus Río Mamoré (RIOMV) was obtained from a small-eared pygmy rice rat (*Oligoryzomys microtis*) trapped in the region where the man worked and was presumably infected [3]. Although RIOMV has not been definitively linked to human disease, fatal HPS has been reported in eastern Brazil putatively linked to viruses (coined Anajatuba and Río Mearim) phylogenetically very similar (94–96% nucleotide homology) to RIOMV [11], [12]. Although RIOMV-infected small-eared pygmy rice rats have been found to date only in the Bolivian departments of Santa Cruz, La Paz, and Beni, and in neighboring Peru, the range of this species, and thus the potential area at risk for RIOMV transmission, is vast, including the Amazon Basin of Brazil and contiguous lowlands of Peru, Bolivia, and Paraguay [13]–[17].

Assuming that the episode of rodent urine falling on the physician's face was the infecting event, we can calculate a precise incubation period of 33–35 days for this case (still with a small range, since the physician did not recall which day during his weekend stay the exposure occurred). This is in keeping with other reported incubation periods for HPS [18].

Four different hantaviruses have now been confirmed in Bolivia: LANV [3], [6], RIOMV [15], Bermejo [19], and Andes [7], [20]. Furthermore, two distinct hantaviruses circulate in the Department of Santa Cruz (LANV and RIOMV) and three in Tarija (LANV, Andes, and Bermejo)(Figure 1). Cases of HPS have also been reported from Cochabamba Department, although no information is available on the specific hantavirus involved [21]. The risk of infection should probably be considered particularly high in the Departments of Santa Cruz, Tarija, and Cochabamba, especially to agricultural workers and others with frequent exposure to rodents.

The prevalence of antibody to hantavirus in Santa Cruz Department was relatively high and consistent throughout the areas studied. Age stratification showed a steady increase in IgG antibody-prevalence with increasing age group, suggesting continuous exposure to hantaviruses over the course of the lives of the population (Figure 2). It is notable that, despite the apparent consistently high rate of exposure to hantaviruses, confirmed cases of HPS have never before been reported from Santa Cruz Department. Possible explanations for this finding include frequent mild or asymptomatic infection not necessitating medical care (as we have documented here in the four IgM antibody-cases), inadequate surveillance or reporting, and misdiagnosis of HPS by clinicians, perhaps due to unfamiliarity with the condition compounded by lack of readily available diagnostic testing for HPS in most hospitals. Similar high antibody prevalence with infrequent reporting of HPS has been reported from Cochabamba Department in Bolivia, as well as from neighboring areas of Paraguay, Argentina, and Chile [2], [7], [20], [22]–[25]. In contrast, data do not suggest frequent mild or asymptomatic transmission of Sin Nombre or other hantaviruses in North America [26]–[28].

**Figure 3:**
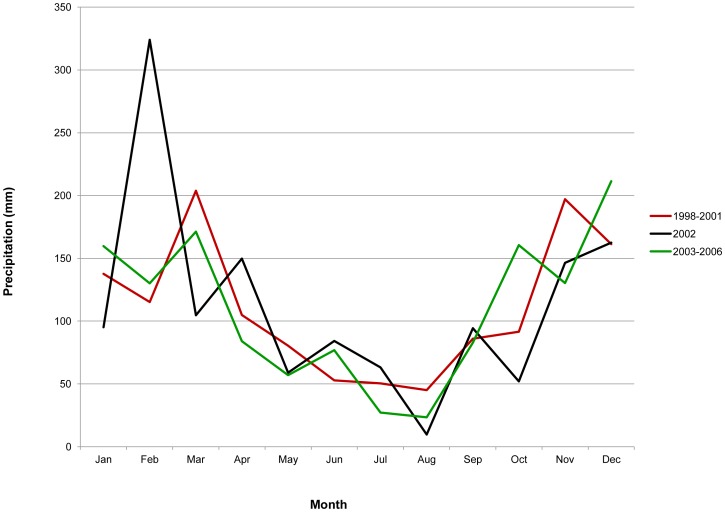
Average monthly precipitation in Santa Cruz de la Sierra, the capital of Santa Cruz Department, comparing the year of the reported hantavirus outbreak (2002) with averages of the preceding and following four years.

The occurrence of six human hantavirus infections (including both PCR- and IgM-positive cases) in two ecologically disparate areas of Santa Cruz Department within a few months may relate to region-wide climatic or ecological influences resulting in increased rodent populations [29]–[31]. Deviations in precipitation or temperature, sometimes associated with effects on flowering and fruiting or seed-set of particular plants known to be significant sources of food for rodents, have been implicated in other hantavirus outbreaks, although the precise relationship remains ill-defined [32], [33]. Interestingly, precipitation in the city of Santa Cruz in the months prior to the outbreak appeared to be particularly heavy in comparison to other years (Figure 3). The cases occurred at the end of the rainy season at a time of cooler temperatures.

The cases reported here occurred in areas of significant anthropogenic perturbation of the landscape (i.e., clearing forest for ranching and sugarcane farming), which is thought to increase the risk of rodent-borne virus infection to humans through the intrusion of opportunistic rodents as well as increased exposure to animal excreta [34]. Interestingly, hantavirus antibody prevalence did not vary among the regions and communities we studied, despite the presumed lower risk in the groups not primarily involved in agriculture. Possible explanations for this finding include the non-random sample and that many of the non-agricultural workers still had significant rodent exposure in and around their homes. Furthermore, it is possible that the rate of exposure varies significantly between the populations, with the antibody prevalence in the more stable urban population representing life-time exposure while the largely immigrant agricultural population may have achieved a similar prevalence in the much shorter time since coming from other, perhaps non-endemic regions. The work reported here was part of an outbreak investigation. Larger and more comprehensive antibody prevalence surveys, including longitudinal studies, systematic rodent surveys with hantavirus testing, and aggressive case surveillance would help elucidate the epidemiology of HPS and the potential risk to humans in Santa Cruz Department.
